# Supplementary figures and images for: A portable brightfield and fluorescence microscope toward automated malarial parasitemia quantification in thin blood smears
Source: PLoS One. 2022 Apr 7;17(4):e0266441. doi: 10.1371/journal.pone.0266441 (PMC8989350; doi:10.1371/journal.pone.0266441)

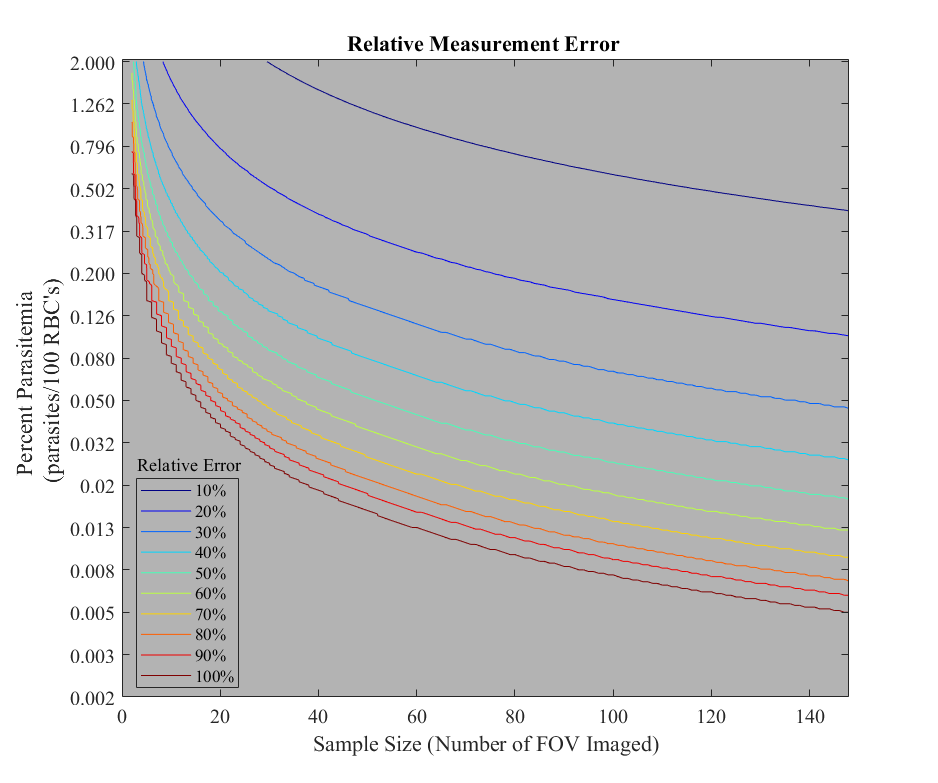

Supplement: S1 Fig — Contour plot of relative measurement error at the edge of the measurement 95% confidence interval for varying parasitemia and sample sizes. Parasitemia is defined as the number of parasites per 100 red blood cells, and the sample size is listed in number of fields of view examined assuming the average of 670 cells per field of view. (TIF) [file pone.0266441.s001.tif]

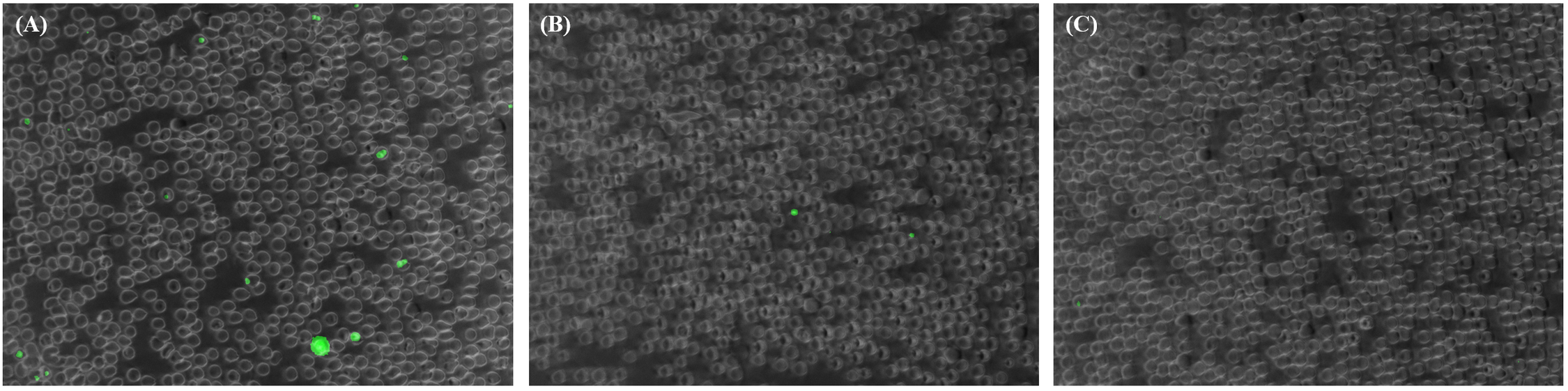

Supplement: S2 Fig — Composite images from portable microscope showing smears with: (A) high 1% parasitemia, 833 cells, 13 parasites; (B) medium 0.1% parasitemia, 1005 cells, 4 parasites; (C) low 0.01% parasitemia, 833 cells, 2 parasites. The brightfield image from each was inverted and linearly contrast enhanced for clarity, and the fluorescent image overlay shows the segmented object pixels overlaid using the green image channel using FIJI ImageJ software. (TIF) [file pone.0266441.s002.tif]
