# Supplementary material for: A portable brightfield and fluorescence microscope toward automated malarial parasitemia quantification in thin blood smears
Source: PLoS One. 2022 Apr 7;17(4):e0266441. doi: 10.1371/journal.pone.0266441 (PMC8989350; doi:10.1371/journal.pone.0266441)
Supplement: S1 Table — (PDF) [file pone.0266441.s003.pdf]

# S1 Table

**Table S1:** Costs of major components in portable microscope prototype.

| Description | # | Price/ea. | Total Price |
|---|---|---|---|
| Nvidia Jetson Nano | 1 | $89 | $89 |
| Sparkfun Stepper Motor #ROB-09238 | 2 | $17 | $34 |
| FLIR Blackfly S 1.6MP Mono | 1 | $315 | $315 |
| Geltech Asphere Lens #355110 | 1 | $98 | $98 |
| Newport Bi-Convex Lens #KBX064 | 1 | $41 | $41 |
| Newport Bio-Convex Lens #KBX043 | 1 | $41 | $41 |
| Thorlabs Asphere Lens #ACL25416U | 2 | $19 | $38 |
| Semrock Filter Set #FITC-LP01-Clinical-000 | 1 | $625 | $625 |
| Osram 523 nm Green LED # LZ1-00G102-0000 | 1 | $5 | $5 |
| CREE 485 nm Blue LED #XPEBBL-L1-0000-00301 | 1 | $2 | $2 |
| | | **Total Cost:** | $1318 |
