# Supplementary material for: A portable brightfield and fluorescence microscope toward automated malarial parasitemia quantification in thin blood smears
Source: PLoS One. 2022 Apr 7;17(4):e0266441. doi: 10.1371/journal.pone.0266441 (PMC8989350; doi:10.1371/journal.pone.0266441)
Supplement: Attachment — Submitted filename: Response to Reviewers-02.pdf [file pone.0266441.s005.pdf]

Response to Reviewers

The authors wish to thank the Reviewers once again, sincerely, for their additional feedback. We believe that the included revisions have addressed all reviewer concerns and have sought to incorporate the feedback in the manuscript.

All reviewer points of feedback are copied herein in standard font, and all author responses are following in italics.

Reviewer #1

I thank the authors for their great effort to address the comments from the reviewers. The revised manuscript has been greatly improved from the first draft.

Two more comments:

1. The units standard used by the authors are not consistent. In Line 164~165, I encourage the authors to change to unit to international standard unit, i.e., mm and kg.

   The units of measurement have been converted to the metric convention.

2. The illumination system of the microscope was housed in the lid of the microscope. When the lid was opened and then closed, will the alignment of the lid with the base affect the image quality? I suggest to add some mechanism along the edge of the lid and base (some sort of kinematic design) to ensure better alignment.

   Although image quality reduction due to misalignment of the illumination system was not problematic during benchtop testing, the potential need for alignment features is acknowledged. During the tests as a part of this work, the rigidity of the microscope case and design of the illumination system which filled an area of the imaging plane slightly greater in size than the field of view prevented issues. The harshness of field testing may necessitate that future design revisions include kinematic features such as tapered female/male cone pairs to ensure optical alignment when the lid is closed. This limitation was noted in the manuscript. (Line 384).

Reviewer #2

This resubmission by Paul Gordon et al. describes the development of a low-cost, portable microscope with brightfield and fluorescence imaging functions. This bimodal imaging capability is helpful in detection of Plasmodium parasites in thin blood smears using relatively low magnification lens. Design and engineering of the microscope prototype are described in great details. The strengths of this study are the novel bimodal microscopy, low cost ~ $1,300, and its compatibility to blood smears prepared on conventional glass slides and inside microfluidic channels. Overall, this paper has its merit, and the writing is fluent and generally clear. Some questions should be clarified by the authors as listed below:

Comments:

1. Was there any attempt to confirm the parasitemia of the Plasmodium cultures using benchtop microscope under higher magnifications? For example, immersion oil 60x or 100x can provide accurate analysis on parasites through early ring to late trophozoite stages. If so, it would help validate the measurements while eliminate the requirement of PCR use and reduce the effects of the possible artifacts of the fluorescence microscopy.

   The parasitemia of the culture was measured using benchtop microscopy under high magnification. This was made more explicit in the Experimental Procedure section (Lines 222-223). However, due to the known differences in parasite contrast and sensitivity of detection when Giemsa and fluorescent stains are used, it was determined to be necessary to utilize a direct fluorescence-to-fluorescence control image to measure the accuracy of the portable system. As previously noted, PCR use would be the better alternative for control measures in future studies.

2. Sample size (number of FOVs) is dependent on the quality of the smears, where only the FOVs of monolayer of cells is collected and analyzed. Although the preliminary results using dilutions of parasite cultures show good agreement between benchtop and portable measurements at high parasitemia, >500 parasites/µl blood. This may be problematic for the intended field use and PoC settings, for real samples with low parasitemia and poor-quality smears.

   The necessity of high-quality monolayer smears is indeed a drawback of microscopy use for parasitemia quantification in general, whether it be this portable system or conventional lab-based microscopy. This drawback was made more explicitly clear. (Line 409). The awareness of this drawback is what prompted the referenced but separate study of thin-smear microfluidic cartridges which were developed alongside this microscope.

3. It is unclear what algorithm was used to differentiate monolayers from multilayers. Was it done manually during microscopy and data collection or retrospectively?

   During this study, monolayer regions of the smears were imaged by manual inspection during data collection. In the future, improved algorithm developments could automatically identify monolayer regions in real-time during imaging. Two possible filtering methodologies would include analyzing edge feature circularity via application of a Sobel filter or the verification of the red-blood-cell spatial frequency in a 2D Fourier Transform. This distinction has been briefly noted in the manuscript. (Lines 245-249).

4. The present imaging analysis method does count all parasites in the smears. Could an algorithm be added to correlate between brightfield and fluorescence images of a same FOV, so that cellular and extracellular parasites can be differentiated?

   Differentiation between intracellular and extracellular parasites could be accomplished using a post-processing algorithm, however the utility was not self-apparent, and it was not included in this work.

5. (minor) Authors removed all relevant descriptions and claims on drug resistance testing. In my opinion, a significant advantage of microscopy method than RDTs in malaria detection is that it can be potentially useful in differentiation live parasites from dead parasites for drug resistance screening. Can you comment would it be possible to implement such function, e.g., addition of

another fluorescence filter? If so, what would be the extra amount of work/cost? I understand the current revision has been made to focus more on the diagnostic applications.

This is a very interesting comment, and one which we spent some time exploring in an alternative line of experimentation that is not included in this work. When imaging "live" parasites, there are several different approaches to differentiating between those that are living and dead. From conventionally fixed thin smears that are dry, all parasites in images are dead at the time of imaging, but clues as to the living population at the time of sampling could be gathered from parasitic morphology and location. However, using the aforementioned microfluidic channel smears, cells are not technically fixed when they are imaged. While this presents its own limitations, it allows for truly living parasites to be examined in great detail, with fluorescently tagged parasites able to be tracked as they move about the smear in real time. The authors believe there is potential utility in this approach for drug resistance monitoring.

6. Fig. 4, please add quantifications of these representative images, e.g., RBC count and parasites count analyzed from both benchtop and portable microscope images.

   Images in Fig 4 were cropped to match the size of shown FOV and number of cells. In the raw source images, the Benchtop images contained 4,275 cells and 44 parasites (1.0%); while the portable images contained 833 cells and 13 parasites (1.6%). The cropped images shown in the figure contain 593 cells. These data have been included in the figure caption.

7. I would appreciate some representative image data of low, moderate, and high parasitemia samples being added, either to the Fig. 4 or in the supporting information.

   A figure has been added to the Supporting Information showing high, medium, and low parasitemia images gathered from the portable microscope. A reference to this figure was added to the "Quantitative Parasitemia Measurement" section (Line 338).

Other comments:

8. 1. Line 310 has a typo "hve".

   The typo has been corrected.
