# Supplementary material for: A portable brightfield and fluorescence microscope toward automated malarial parasitemia quantification in thin blood smears
Source: PLoS One. 2022 Apr 7;17(4):e0266441. doi: 10.1371/journal.pone.0266441 (PMC8989350; doi:10.1371/journal.pone.0266441)
Supplement: Attachment — Submitted filename: Response to Reviewers.docx [file pone.0266441.s004.docx]

Response to Reviewers

*The authors wish to sincerely thank the Reviewers for their excellent and thorough feedback, especially as it relates to clarifying the purpose of the work and incorporating it into the broader field. The authors also thank the editors and reviewers for their patience as revisions have been completed.*

*Overall, there have been many minor revisions that should improve the robustness of the work and the clarity of communication for the work that has been done. The primary changes were to emphasize that the work was meant to be a technical description of a new technology rather than a description of a diagnostic test itself. Nearly all comments were accepted and implemented in the paper.*

*All reviewer points of feedback are copied herein in standard font, and all author responses are following in italics.*

**Reviewer #1**

1. Abstract - Please provide more information regarding study methodology in the Abstract. What types of samples were analysed and how were they prepared (i.e.thin smears, fluorescently stained, laboratory culture samples), how many samples were used, specific results (level of correlation) and reference standard used (visual or digital analysis of samples?). Also, avoid speculation in the Abstract which are not related to the research findings (e.g. here compatibility with machine learning solutions, this is not explored in the study).

- *The speculative elements of the abstract were removed, and statements regarding sample type and number were included to address methodological shortcomings.*

2. Application and Potential Impact – I would consider moving this section (or the relevant information from this section to the Discussion)

- *The second paragraph of the section was moved to Discussion, and the first paragraph was merged into the Introduction section.*

3. Ethical statement - Although the submission information states that no ethical information is required, I would considering adding this information (as well as why no information is required) as a short statement to the manuscript body, as this is an article exploring a medical diagnostic technology.

- *A short statement describing the purpose, disclaimer, and lack of conflicts of interest for the work was added prior to the Acknowledgements section.*

4. I noticed your interesting previous work in this field from recent years (e.g. (<https://spie.org/Publications/Proceedings/Paper/10.1117/12.2510593?print=2> and <https://www.ncbi.nlm.nih.gov/pmc/articles/PMC6997630/>)
   • Is the same system used? Presumably these findings relate in some way, I would consider including a reference or brief summary on your previous work in this field. Have you updated the hardware, made technical adjustments or addressed other challenges?

- *Two sentences were added to the Microscope Configuration paragraph (along with citations) that address the progression of system design to its current state.*

5. Introduction (and Discussion) - Interestingly, we have published a study last year describing a very similar, also quite low-cost system with quite comparable imaging performance (<https://journals.plos.org/plosone/article?id=10.1371/journal.pone.0242355>). As you no doubt are aware, there are a number of other similar systems, as several other papers have investigated systems for field digitization of e.g. fluorescently stained samples (e.g. already in 2009 by Breslauer et al.), followed by e.g. CyScope and the Parasight system. I would consider briefly reviewing what the findings of previous work in the field have been to provide the reader with perspective and better understand the context and implications of your work. This is especially important as you state that this is the lowest cost similar system (the price seems to be approximately comparable to certain other solutions, although understandably it is difficult to establish a final price based on a prototype)

- *An additional paragraph giving an overview of portable microscopes along with predicate fluorescence imaging systems has been added to the introduction. The discussion has been modified to qualify the remarks about the cost novelty and to better address the unique contributions of the described system to this area of research (use of singlet aspheric objective lens with long working distance, potential compatibility with cartridge based smears, and monochromatic visible wavelength illumination).*

6. Page 6, line 116 - Please define abbreviation SNR (signal-to-noise ratio).

- *The abbreviation has been clarified.*

7. Line 151, page 8 – It seems Figure 2 and 3 have accidentally been interchanged (also e.g. Line 220, page 11)

- *The image reversal has been corrected.*

8. Lines 187 – 201 - I would appreciate to read some more details about the operation of the system. How is the sample scanning performed – is the sample translated automatically or by manual adjustment of the sample to change FOV before digitization of the next one?

- *Current device control is primarily manual due to the need to account for variances in smear thicknesses across each slide. The authors believe this is a control problem that can be easily overcome in future product development stages and does not significantly affect the novelty of the system. More descriptive language was added to the experimental procedure section to elucidate the current state of device control.*
  - How was the field of view (FOV) with the monolayer region selected?
  - *Slides were placed on the stage so that the FOV was biased toward one side of the monolayer region, and the slides were advanced linearly through the monolayer region. Visual identification of cell separation on the display was used to bypass non-monolayer FOV’s. While this manual process is less than ideal, it is, again, a feature that can be addressed with more refined control software and does not detract from the novelty of the optical system employed.*
  - How large are the areas which are digitized (is the complete slide digitized)?
  - *The slide is not completely digitized. In these tests, the microscope scans through a linear section of the smear monolayer. Due to the wider FOV compared to 100x microscopy, this was found to image enough cells to allow for the data collection as presented in the paper. If it is found in future iterations that more cells must be imaged on each slide, vertical scanning could be added (in addition to the lateral scanning already integrated).*
  - Approximately how many RBCs and parasites were on average analyzed per slide (this is perhaps included in the MATLAB library online, but I would consider this information quite important to interpret the results, and therefore include some information also in the manuscript). If only a small image region is analyzed, the results might differ quite a lot based on the area which is analysed.
  - *A table displaying the total counts of all cells, FOV, and objects has been added to the results section of the paper.*
  - Line 194 – what is an “adequate number of images” and how was this amount decided? Did it differ between samples?
  - *An “adequate number of images” was a vague description for the total usable number of FOV from each smear, which varied. This language has been corrected in the description and more precisely represented by the inclusion of new Table 1.*
  - What was the turnaround time for digitization and analysis of one slide?
  - *The turnaround time for digitization and analysis was not captured during these experiments. Capture of two images for each FOV required approximately 30 seconds, which was primarily derived from the time needed to input each translation and capture command. Data analysis was conducted retrospectively and thus could not be measured in a way that is representative of the device’s potential eventual use. Statements clarifying these points were added to the methods and results sections to prevent reader confusion.*

9. Methods and Results - How many thin smears did you analyze, i.e. what was the sample size and how was it decided? Was a single slide analyzed for each parasitemia dilution level? How were the statistical calculations performed, what was the level of significance and what software was used?

- *In total, 16 thin smears were analyzed. MATLAB software was used to calculate the relationships between parasitemia, sample size, and width of the 95% confidence interval of parasitemia measurement, which can be mapped in a 3-dimensional solution space as a contour plot of relative measurement error. The statistics were calculated using the Clopper-Pearson method for approximating the confidence intervals for rare events in a binomial distribution. This approach was chosen to contextualize the statistical significance for any measurements gathered depending on the number of available monolayer FOV per smear imaged.

  Given that the current intended use of the microscope is for parasitemia quantification for the purpose of informing diagnostic decisions between high-risk and low-risk cases, the desired sample sizes were determined to achieve statistical significance at these admittedly high parasitemias. Ultimately, the number of cells imaged for each slide was constrained by the limit of the quality of the lateral section of FOV’s in each, which yielded, on average, over 12,000 cells per sample, enough to result in a maximum relative error of 50% at 0.1% parasitemia and 90% at 0.05% parasitemia. All measurements of the target parasitemia dilutions were conducted in duplicate, except for 0.75% (due to experimental error resulting in loss of sample, but which was included to provide a total picture of the data gathered).

  A brief statistics statement has been added to the Experimental Procedure section, and a more expansive statistical treatment with a brief literature survey and figure showing the results of the Relative Error contour plot has been added to the Supplemental Information of the paper.*

10. Methods - Although I understand that the parasitemia dilution levels were available from the preparation of the samples, these are apparently not used as a reference (e.g. Fig 5). It is a bit unclear to me what the reference standard is, e.g. is the Benchtop Parasitemia Measurement determined by manual (visual) analysis of the digital sample with the high-end microscope, or is the same digital image analysis software used, similarly to the low-cost solutions? If manual assessment of samples was used, how was the parasitemia level determined?
    - *The parasitemia dilution levels were created based on the overall culture parasitemia (1.2%), which was determined using manual counting of parasites and red blood cells from three separate “Gold-Standard” Giemsa-stained thin smears. This counting was performed using ImageJ for digitized images gathered using oil-immersion microscopy on the benchtop high-quality system. The same object segmentation and thresholding procedures were used to process images collected on both the reference benchtop system and portable microscope, although different size and intensity thresholds were used for each due to differences in magnification and illumination between the systems. This method has been clarified in the Experimental Procedure and Quantitative Parasitemia Measurement sections.*
11. Methods - What samples were used to develop the software used for analysis of the samples, i.e. what samples did you use to determine the threshold settings to separate e.g. artefacts from parasites? Were these samples images from the same samples you analyzed, or completely different samples? Preferably the training data should be separated from the validation data.

- *Development of the image processing techniques was done using images of the control culture smear gathered using the portable and benchtop fluorescence microscopes. The processing was thus developed on data gathered and analyzed as a part of the study, however it was tuned using only the control sample and not the dilutions which were later analyzed, giving some degree of separation between algorithm development and implementation. The limitations of this slight overlap have been noted in the Limitations section.*
  - During our similar work, we found one unfortunately quite significant challenge to be the separation of artefacts from parasites by analysis of only the fluorescent samples, especially in conventional thin smears collected in the field (see e.g. S3 Fig <https://journals.plos.org/plosone/article?id=10.1371/journal.pone.0242355#sec018>, which represents a field sample). By brightfield analysis of Giemsa stained sample using high-power (60-100x) microscopy, it is possible to more reliably confirm the parasites, but e.g. the spatial resolution in Figure 4 seems to suggest that this would be quite challenging to do using the levels of magnification here. How did you confirm e.g. that the fluorescent signals with lower intensities that were classified as artefacts were not trophozoites? Although this is briefly discussed on page 12, perhaps e.g. image examples here could illustrate the difference between artefact and parasite more clearly.
  - *Perhaps the most significant limitation of the study conducted is the lack of qPCR controls for comparison of all microscopy techniques used (brightfield high-magnification, benchtop fluorescence, and portable fluorescence), as noted by Reviewer #2. In hindsight, this would have been the most instructive way to parse the difference between what fluorescent features are truly artefacts or are parasites that did not uptake the Giemsa stain in a way that made them as differentiable as did the fluorescent stains. This limitation is noted in the discussion section to attempt to address the challenge.*

12. Methods - How were digitized images stored – locally or uploaded to the internet? What is the file format and level of compression, and are the images saved as individual FOVs or stitched to whole-slide images? A figure demonstrating a “scanned sample” would perhaps illustrate this. Can the system operate in regions without network access?

- *All files were saved locally to a portable USB flash drive in raw format, then transferred to a laptop computer for retrospective data analysis. In this way, the system is operable without any network access. This has been noted in the experimental procedure section.*

13. Methods - As the system is aimed for rural, low-resource regions, can the system operate on battery power or does it require a stable power source? Naturally, this could likely of course be solved e.g. by using an external battery

- *The system could be adapted to utilize battery power if desired. The total approximate operating wattage of the system is ~ 18W, which can easily be supplied by a portable battery pack. The reliance on central power for recharging would be theoretically similar to that necessary to charge a laptop computer between uses. This has been noted in the prototype construction section.*

14. Methods - Is the staining technique described here possible to perform in rural settings with access only to basic laboratory equipment, or would it need to be altered to be usable in low-resource environments?

- *The staining technique could be used in remote laboratory settings, with the major limitation being that use of any impure water source for stain dilution or rinsing could cause fluorescent artifacts to appear in samples. Slide staining is similar in difficulty to Giemsa staining, although samples must be allowed to dry in a relatively dark environment. The largest limitation to the staining procedure is the need to store fluorescent stain – Acridine Orange, which operates in comparable fluorescence bandwidths, has been vetted for stability at room temperatures, although to the author’s knowledge SYBR-Green1 has not been. These considerations have been included in a new Limitations section added to the discussion.*

15. Line 264, page 13 - “Preliminary results” - I would consider rephrasing to e.g. “early results” or clarify why these are preliminary. How do these results relate to your previous work (see earlier comments)?

- *The language of “early results” was adopted in the discussion, and a more explicit description of what this means was given in the new Limitations section. A final statement summarizing the improvement made above previous prototypes was also added into the final Conclusions paragraph.*

16. Line 267, page 13 – As you have implicated, a detection level of 0.2 is quite low for clinical utilization and does not e.g. allow the ruling out infections. Please clarify how this is “sufficient to inform most diagnostic decisions” (i.e. in rural setting clinical malaria diagnostics, perhaps one of the most important clinical questions is the differential diagnostics of malaria vs other conditions).

- *The “diagnostic decision” was meant to refer to the differentiation of high and low risk patients among a population that has already been screened using RDT’s. As stated in other parts of the paper, the microscope is not intended for use as a screening tool due to the inherent limitations of achieving sensitive detection in thin smears, especially when compared to the proliferation of rapid diagnostic tests. The statement referenced was modified to clarify the intended use of the device and the relationship to the demonstrated dynamic range.*

17. Line 288, page 14 - Considering moving Table 1 to the Methods section (description of the system) or e.g. Supplementary Information and avoid presenting new information in the Discussion.

- *The table was moved to the Supplemental Information section, and the corresponding paragraph was adjusted to be only a discussion of the comparison of cost to other comparable systems. Reference to the supplemental information section was added to the Methods section.*

18. Line 290, p. 15 - “Results of this study show the feasibility of a method for quantitative parasitemia measurement while away from a centralized microscopy laboratory using an automated, low-cost bimodal microscope optimized for the specific task”. I do not agree that the results suggest this. The reason is that the sample are neither collected, nor prepared (to my understanding) while away from a centralized laboratory. Although the samples are thin smears, I would consider it quite unlikely to be directly comparable to thin blood smears collected in field settings. To my understanding, the results seem to suggest instead that the imaging performance of the presented system seems to be sufficient for determination of the parasitemia level in moderate- to high-level samples, which are prepared in laboratory settings. I therefore think it is premature to draw conclusions regarding usage especially at more remote points of care. I would consider rephrasing this to e.g. instead state that the system is designed for usage at the point of care.

- *The language has been clarified to better represent that more work must yet be done to show feasibility outside of a controlled laboratory environment and to again acknowledge the limitations of the device in the Conclusions section.*

19. Discussion – Please further discuss the strengths and weaknesses of the study, as well as the challenges required before clinical implementation. E.g. although it is stated that the technology could be combined with RDTs for diagnostic applications, it should be emphasized more clearly that the detection level is quite high and needs to be improved to allow confirmatory diagnostics.

- *The Discussion section was broken out into sections for “Advantages & Novelty” as well as “Limitations” and “Conclusions”. The high detection limit is more explicitly stated in the limitations section.*

20. Figure 1 – Please include size of the system in the image (width, length, height) and weight. This could be included in the Methods section also.

- *The dimensions of the system have been included in Fig 1, and the overall device weight was added into the Methods section alongside the device dimensions.*

21. Figures - Although the data from all findings are made available, is any image data available (e.g. illustrating the detected parasites in e.g. a low-, compared to a high-level parasitemia sample? This would make the analysis of the samples more understandable also for non-technical readers, such as clinicians.

- *A figure showing images of FOV from high and low parasitemia samples has been added to the Supplemental Information, and a statement describing it has been added to the Results section.*

**Reviewer #2**

1. The main issue of this study to me is the fact that the authors draw their conclusions based on a relatively small non-blinded laboratory-based experiment without testing the device in field settings and clinical samples.

- *The language throughout the work has been modified to reflect that the study is intended to represent a prototype technology and emphasize that the purpose of the work is the vetting of optical mechanism design, not a robust clinical diagnostic investigation requiring blinded field setting work.*

2. How do the microfluidic cartridges work (these would be a major advantage compared to the use of thin slides as to create a good thin slide requires some skill)

- *The microfluidic cartridges operate by forcing pre-stained blood cells in liquid phase through a small, flat channel in a hydrophilic cartridge. The channels are created by molding the appropriate features into a modified hydrophilic PDMS polymer, then bonding this channel to a glass or plastic substrate. Full details of the cartridge design are referenced in the paper, but are not included in this work as they have been previously published elsewhere.*

3. A blinded study using spiked samples

- *While blinding the study would indeed make the diagnostic implications of the study more robust, it was felt to be unnecessary at this time for the purposes of demonstrating the technical novelty of the proposed system. Future studies that propose to examine the diagnostic performance of the system in a clinical setting would be blinded.*

4. A blinded study using clinical samples

- *Clinical samples were unfortunately unavailable at the time of the study due to travel restrictions imposed by institutional and political authorities.*

5. Preferably a time-series before and after treatment

- *Future clinical studies would be likely to include time-series data when the device can be tested in a setting where treatment is administered.*

6. Preferably a field study performed in field settings to see how it copes with differences in temperature, humidity, dust etc.

- *The lack of testing in a field environment is indeed a limitation of the current methodology. These limitations have been added to the discussion section of the paper.*

7. Second, I think the manuscript should focus more on the experiment itself and the results of the experiment. At the moment the only explicit data shown as to how it functions compared to other techniques is a figure to compare images made by bench-top microscope and the device, and one linear regression analysis.

   This is why I suggested a major revision. Personally, I would like to see additional experiments done before these conclusions are drawn. If the authors would adjust the conclusions to better reflect the actual data presented here a minor revision would be justified. I think that the device has potential, but I also think that it's potential is very limitedly shown in the current data.

- *The paper has undergone significant minor revisions to scale back the conclusions drawn and further emphasize that the novelty proposed is related to the optics and technical specification of the imaging system rather than proving outright that diagnosis is accomplished in these experiments.*

8. A baseline false-positive of 10.000 p/uL is a very high baseline, limiting the usefulness of this technique as it is presented at the moment, and should be acknowledged as such. Particularly taken into consideration that RDT’s have to be able to detect parasitemia at 200 p/uL and that field microscopy is generally able to detect parasitemia counts lower than that

- *These limitations have been explicitly addressed in the results and discussion sections of the paper. There has also been a more extensive statistical treatment presented that briefly explores the challenges associated with achieving low limits of detection with thin smear microscopy. Such limitations are stated and the fact that the device is not intended to achieve a low limit of detection is also stated.*

9. The experiment was not blinded which should be explicitly stated.

- *A statement has been added to the Methods section to explicitly call out that the tests are not blinded.*

10. Samples were not tested in duplicate

- *All samples were tested in duplicate, with the exception of the initial culture parasite concentration and 0.75%, whose duplicate was lost during experimentation. A table giving the total numbers of smears, FOV’s, cells, and fluorescent objects was included to elucidate these points.*

11. Parasitemia levels in the dilutions were assumed rather than tested

- *Although parasitemia dilutions were assumed, the same samples were measured using both systems. This allows the correlation of measurements between the two systems to be preserved, although it admittedly lowers confidence in the absolute accuracy of the results. The lack of a qPCR reference as an absolute control has been noted in the limitations section of the manuscript.*

12. The manuscript places emphasis on technical details on how the portable microscope was designed. As a result, it can be challenging to read for a broader audience.

- *Several technical sections of the manuscript have been rewritten in an attempt to make the content more accessible to broader audiences. The subject matter of the paper is inherently technical in nature, however, and cannot be fully reduced in complexity without sacrificing the novelty of the work, notably specific details of the optical systems that confer new functionality to the device above that which has been previously shown in the literature.*

13. It would be useful if the authors would consistently provide both percentage infected RBC and p/uL or choose one of the two and leave out the other. Right now both are used interchangeably which makes it more difficult to compare

- *The use of p/uL in the paper has been removed, except in the introductory sections where an overview of parasitemia is broadly given. Parasitemia measurements are thereafter either presented as a simple percentage or as per 100 RBC’s, which is more technically correct if extracellular parasites are included in the count. The distinction in this notation has been added to the results section of the paper.*

14. To me it seems like there are a lot of methods in the results section and some new data (e.g. the cost overview as well as the statement that the LOQ was 10.000 p/uL) in the discussion which I found somewhat confusing, though it is a minor remark.

- *The cost data has been removed from the discussion section and either moved to the Methods or Supplemental sections of the paper.*

15. Abstract: In my opinion the authors should be careful with their hypothesis that the technology may be useful in drug resistance screening. First of all, the technique itself does not screen for resistance but rather assesses efficacy of therapy. Second, the current study does not provide data to support this claim as they did not test follow-up, nor any clinical samples. In this, the technique is no different from any other which can quantify parasitemia.

- *All references to screening for drug resistance have been removed from the work. A singular statement regarding the possible use for parasitemia monitoring post-treatment has been included in the discussion section.*

16. Introduction:
    a. Line 57; low parasitemia counts do not necessarily correspond with mild disease and vice versa; I would nuance this statement.
    - *The statement has been nuanced to clarify that high parasitemia and severe disease are not equivalent.*
    b. Line 66: I would nuance this by adding that in many labs data are corrected for bloodcell counts as obtained by hemocytometry.
    - *A qualifying statement indicating this practice has been added to the paragraph.*
    c. It would be useful to add the aim of the current study to the introduction (the current study describes …..) and not just the ultimate goal of the technique that is being developed (line 60 onward). This makes it easier for the public to screen what they are about to read which was a lot more technical than I had anticipated from the title and abstract.
    - *A brief statement of this nature has been added to the beginning of the introduction.*
17. Methods:
    a. Please describe how many samples were tested and what their parasitemia level was according to the culture. Please state how these relate to the portable microscope results
    - *A table was added to the results section that details the sample sizes and results of all measurements in relation to the parasitemia dilution from the culture. A discussion of the discrepancy between parasitemia values found using Giemsa stained and fluorescence stained was added to the Limitations section of the paper.*
    b. Please describe in more detail how test results were interpreted (automated or not) and who interpreted the results (same person for both tests?)
    - *Several sentences addressing this concern were added to the Results section. The test results were interpreted by the same person for both tests. The data used to tune the parameters for the object segmentation algorithms was manually set using data from the full-concentration culture smear as reference, then all dilutions were automatically processed using the parameters that were preset.*
    c. The FOV without monolayer cells excluded: did this significantly decrease the number of fields included in the analysis? Please provide numbers on how many fields were captured per slide and how many were excluded. Please state in the discussion how this would affect the results
    - *Numbers of FOV captured per slide were included in the sample size table added to the result section. Due to the nature of the thin smears being analyzed, each slide contained far more possible FOV that were not monolayer, than that which were. Thus, the number of FOV able to be analyzed is related to the quality of the smear, not the microscope or analysis portions of the study. This topic was added to the discussion section of the paper.*
    d. I wonder if logistic regression comparing test results of two tests which were (as I assume) interpreted by the same researcher is an appropriate choice. If possible, it would be nice to compare with qPCR instead.
    - *qPCR would be a more descriptive and accurate reference standard to compare to both the benchtop and portable system. The lack of this control was a flaw in study design and has been noted in the limitations section of the paper.*
18. Results: It would be nice to see an image of how the results are presented to the technician.

- *At this stage of system development, data are processed retrospectively on a laptop after data have been collected. There is not currently a user interface that directly shows results to the technician, as that work requires further input from clinical stakeholders to properly design in later stage product development efforts.*

19. Discussion: I am missing a limitations section

- *A limitations section has been added to the Discussion.*
